# DNA Methyltransferase Regulates Nitric Oxide Homeostasis and Virulence in a Chronically Adapted Pseudomonas aeruginosa Strain

**DOI:** 10.1128/msystems.00434-22

**Published:** 2022-09-15

**Authors:** Shuhong Han, Jihong Liu, Mianhuan Li, Yizhou Zhang, Xiangke Duan, Yingdan Zhang, Hao Chen, Zhao Cai, Liang Yang, Yang Liu

**Affiliations:** a Medical Research Center, Southern University of Science and Technology Hospital, Shenzhen, China; b School of Medicine, Southern University of Science and Technology, Shenzhen, China; c Department of Clinical Laboratory, Shenzhen Third People's Hospital, Second Hospital Affiliated to Southern University of Science and Technology, National Clinical Research Center for Infectious Diseases, Shenzhen, Guangdong, China; d Shenzhen Third People’s Hospital, Second Hospital Affiliated to Southern University of Science and Technology, National Clinical Research Center for Infectious Disease, Shenzhen, China; e Shenzhen Key Laboratory of Gene Regulation and Systems Biology, Southern University of Science and Technology, Shenzhen, China; Génomique Métabolique, Genoscope, Institut François Jacob, CEA, CNRS, Université d’Évry, Université Paris-Saclay

**Keywords:** *Pseudomonas aeruginosa*, DNA methyltransferase, denitrification, virulence, bacterium-host interactions, DNA methylation

## Abstract

Opportunistic pathogens such as Pseudomonas aeruginosa adapt their genomes rapidly during chronic infections. Understanding their epigenetic regulation may provide biomarkers for diagnosis and reveal novel regulatory mechanisms. We performed single-molecule real-time sequencing (SMRT-seq) to characterize the methylome of a chronically adapted P. aeruginosa clinical strain, TBCF10839. Two *N*^6^-methyladenine (6mA) methylation recognition motifs (RCC**A**NNNNNNN**T**GAR and TRG**A**NNNNNN**T**GC [modification sites are in bold]) were identified and predicted as new type I methylation sites using REBASE analysis. We confirmed that the motif TRG**A**NNNNNN**T**GC was methylated by the methyltransferase (MTase) M.PaeTBCFII, according to methylation sensitivity assays *in vivo* and *vitro*. Transcriptomic analysis showed that a Δ*paeTBCFIIM* knockout mutant significantly downregulated nitric oxide reductase (NOR) regulation and expression of coding genes such as *nosR* and *norB*, which contain methylated motifs in their promoters or coding regions. The Δ*paeTBCFIIM* strain exhibited reduced intercellular survival capacity in NO-producing RAW264.7 macrophages and attenuated virulence in a Galleria mellonella infection model; the complemented strain recovered these defective phenotypes. Further phylogenetic analysis demonstrated that homologs of M.PaeTBCFII occur frequently in P. aeruginosa as well as other bacterial species. Our work therefore provided new insights into the relationship between DNA methylation, NO detoxification, and bacterial virulence, laying a foundation for further exploring the molecular mechanism of DNA methyltransferase in regulating the pathogenicity of P. aeruginosa.

**IMPORTANCE** Pseudomonas aeruginosa is an opportunistic pathogen which causes acute and chronic infections that are difficult to treat. Comparative genomic analysis has showed broad genome diversity among P. aeruginosa clinical strains and revealed their different regulatory traits compared to the laboratory strains. While current investigation of the epigenetics of P. aeruginosa is still lacking, understanding epigenetic regulation may provide biomarkers for diagnosis and facilitate development of novel therapies. Denitrification capability is critical for microbial versatility in response to different environmental stress conditions, including the bacterial infection process, where nitric oxide (NO) can be generated by phagocytic cells. The denitrification regulation mechanisms have been studied intensively at genetic and biochemical levels. However, there is very little evidence about the epigenetic regulation of bacterial denitrification mechanism. P. aeruginosa TBCF10839 is a chronically host-adapted strain isolated from a cystic fibrosis (CF) patient with special antiphagocytosis characteristics. Here, we investigated the regulatory effect of an orphan DNA MTase, M.PaeTBCFII, in P. aeruginosa TBCF10839. We demonstrated that the DNA MTase regulates the transcription of denitrification genes represented by NOR and affects antiphagocytic ability in bacteria. *In silico* analysis suggested that DNA methylation modification may enhance gene expression by affecting the binding of transacting factors such as DNR and RpoN. Our findings not only deepen the understanding of the role of DNA MTase in transcriptional regulation in P. aeruginosa but also provide a theoretical foundation for the in-depth study of the molecular mechanism of the epigenetic regulation on denitrification, virulence, and host-pathogen interaction.

## INTRODUCTION

Pseudomonas aeruginosa is a Gram-negative, facultative anaerobic bacterium widely distributed in various environments, such as soil and water. It is a leading nosocomial pathogen that causes severe infections in immunocompromised patients, such as patients with cystic fibrosis and ventilation-associated pneumonia (1, 2). It has a relatively large (5.5- to 7-Mb) and diverse genome encoding a variety of regulators and virulence factors, which enable extensive adaptability to environmental changes (3). Infections caused by this bacterium are difficult to eradicate due to its intrinsic resistance mechanisms and large sets of virulence products (4). Previous genomic analysis studies have revealed many adaptive evolutionary traits of P. aeruginosa during chronic infections, where critical mutations in regulator-encoding genes such as *lasR*, *mucA*, and *rpoN* often reshape the bacterial physiology, resistance, and virulence (1). However, there are only a few studies about how epigenetic control such as DNA methylation is involved in regulation of P. aeruginosa virulence mechanisms (5). New insights into P. aeruginosa epigenetic regulation could facilitate the development of novel diagnosis as well as therapeutic approaches.

Epigenetic control in bacteria is mainly achieved through the activity of DNA methyltransferases (MTases). Bacterial DNA MTase transfers a methyl group from the donor, *S*-adenosine-l-methionine (SAM), to a specific position on the target base to form different modifications (6). There are three main types of bacterial DNA methylation modifications, with *N*^6^-methyladenine (6mA) being the most common type, while the other two types are *N*^4^-methylcytosine (4mC) and 5-methylcytosine (5mC). These modifications protrude into the major groove of the DNA double helix, affecting the interaction of proteins like transcription factors and DNA repair and replication enzymes. The methylated bases could affect protein interactions negatively through steric hindrance or positively by engaging specific proteins evolved to recognize and bind to methyl groups (7). DNA MTase originates from the restriction modification (R-M) system, which is the early defense mechanism of bacteria against invasive foreign DNA. DNA MTase usually works together with the other member of the R-M system, restriction endonuclease (REase), to cut foreign DNA at specific sites (action of REase) and methylate these sites to protect its own genome from being cut (action of MTase) (8). In addition, the discovery of orphan DNA MTases (MTases without coupled REases) shows that DNA MTase can function as a gene expression regulator on its own (9). Phase-variable R-M systems have also been found to flexibly regulate virulence in many bacteria (10). Sequencing techniques such as single-molecule real-time sequencing (SMRT-seq) enable the detection of methylation signals and allow the characterization of prokaryotic genome-wide epigenetic regulation (11). Cumulative studies revealed the function of bacterial DNA MTase and the involvement of DNA methylation modifications in intracellular physiological processes (9, 12, 13). Many bacterial pathogens have been shown to regulate gene expression through epigenetic mechanisms during host colonization and infection (14–16).

There have been only a few studies on the role of DNA methylation in gene expression regulation in P. aeruginosa. Doberenz et al. (5) discovered an adenosine DNA MTase encoded by *hsdM* in model strain PAO1 and identified its methylation site in the promoter region of the noncoding small RNA *prrF1*. Loss of the MTase or methylation site leads to increased *prrF1* transcription, which in turn regulates the iron reserve reaction and weakens the virulence in Galleria mellonella (5). Huang et al. found that 6mA methylation in the promoter of an endotoxin A regulating gene, *toxR*, in clinical isolate PB350 may cause higher expression of OpdQ, a member of the OprD porin family for the efflux of β-lactam antibiotic imipenem (17). Further characterization of the DNA methylation modification mode of clinical P. aeruginosa strains can expand the understanding of the regulation network of gene expression at the epigenetic level and provide new insight into the diagnosis and treatment of clinical infection.

P. aeruginosa clinical isolate TBCF10839 (referred to here as TBCF) was isolated from a cystic fibrosis (CF) patient. This strain evolved for decades in CF patients and has gained the capacity to resist phagocytic cells, although the underlying mechanism is not completely clear (18). In this study, we used SMRT sequencing to provide the first insight into DNA methylation pattern of TBCF. We predicted an orphan MTase and identified its methylated sites and target sequence motifs throughout the genome. The DNA methylation target motifs were confirmed by liquid chromatography-tandem mass spectrometry (LC-MS/MS) analysis, as well as SMRT sequencing of an MTase gene knockout mutant strain. Transcriptomics analysis indicated that DNA methylation positively regulates the expression of nitric oxide reductase (*nor*) genes. Such DNA methylation was also shown to increase both the intracellular survival rate of TBCF in NO-producing macrophages and virulence in G. mellonella. Our results emphasize the role of DNA methylation as a regulator of gene expression in a clinical P. aeruginosa strain and illustrate its involvement in the regulation of phenotypic traits important for bacterial adaptation to host environments during establishment of infection.

## RESULTS

### Methylome analysis and DNA MTase identification of P. aeruginosa TBCF.

To characterize the methylome and identify DNA MTase in TBCF, SMRT-seq was applied to obtain whole-genome sequence and methylation information. The quality of SMRT-seq reads was assessed. The polymerase read-to-subread ratios (PSR) for TBCF and TBCFΔ*paeTBCFIIM* are 0.297 and 0.324, respectively. The SMRT-seq subread *N*_50_ values for TBCF and TBCFΔ*paeTBCFIIM* strain are 13,255 bp and 22,253 bp, respectively. Five MTase genes were predicted in the TBCF genome based on REBASE analysis. For the 3 MTases belonging to type II R-M systems, the methylation motifs predicted by REBASE were not detected in our SMRT-seq data. Only the two MTases belonging to type I R-M systems, M.PaeTBCFI and M.PaeTBCFII, were in perfect agreement with the REBASE predictions, i.e., corresponding to the modifying motifs RCCANNNNNNNTGAR and TRGANNNNNNTGC (underlining indicates modification sites) respectively. The GC contents around the R-M system PaeTBCFI- and PaeTBCFII-encoding loci are 56% and 54%, respectively, which are much lower than throughout the TBCF genome (66%). Both MTases have nearly balanced strand-specific methylation patterns and are for the most part fully methylated on both strands of the recognition motifs. There are more methylation motifs of M.PaeTBCFII (1,426) than of M.PaeTBCFI (396) in TBCF genome (Table 1; also, see Fig. S1 in the supplemental material). In this project, we chose the putative orphan (i.e., with no associated endonuclease) DNA MTase M.PaeTBCFII as the main research object.

**TABLE 1:** DNA MTases and methylation recognition motifs in TBCF^*a*^

MTase	Abbreviation	Recognition motif^*b*^	Modification site^*c*^	No. detected^*d*^	No. in genome^*e*^	Fraction^*f*^
M.PaeTBCFI	AP	RCC**A**NNNNNNN**T**GAR	4, −4	375, 388	396, 396	0.947, 0.980
M.PaeTBCFII	CP	TRG**A**NNNNNN**T**GC	4, −3	1,360, 1,370	1,426, 1,426	0.954, 0.961

a Both MTases are restriction-modification system type I.

b The modification sites in the positive strand are in bold and underlined; the modification sites on the complementary strand are in bold only.

c Negative values represent methylation sites on the complementary strand.

d No. of methylated motifs detected.

e No. of motifs in the genome.

f Ratio of motifs with methylation.

10.1128/msystems.00434-22.3 FIG S1 Restriction-modification systems predicted in TBCF. (a) Five R-M systems were predicted in TBCF, including 2 type I R-M systems and 3 type II R-M systems. (b) Structures of two DNA methyltransferases (M.PaeTBCFI and M.PaeTBCFII [AP and CP, respectively]), which belong to type I R-M systems. M.PaeTBCFII is an orphan enzyme. The prediction was performed using REBASE (41). Methyltransferase genes are shown as thick blue arrows, specificity genes as green arrows, and restriction enzyme genes as red arrows. Flanking, non-R-M genes are shown in grey. The characteristic sequence motifs dppy and fgg (53) are shown on flags above the schematics of the methyltransferase genes. The coordinates beneath each schematic are from NEBC15646. Download FIG S1, PDF file, 0.2 MB. Copyright © 2022 Han et al. 2022 Han et al. https://creativecommons.org/licenses/by/4.0/ This content is distributed under the terms of the Creative Commons Attribution 4.0 International license.

### *In vivo* verification of putative MTase activity and specificity.

**FIG 1:**
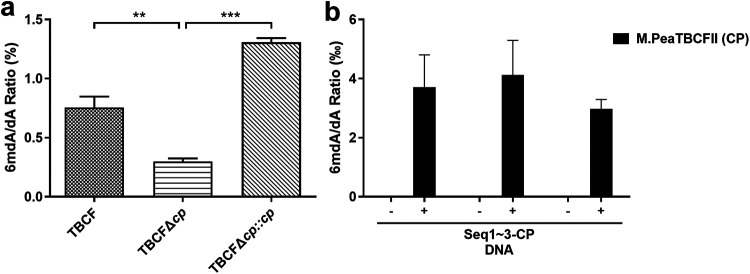
DNA MTase enzyme activity analysis using LC-MS/MS. (a) *In vivo* verification. The 6mdA/dA ratio in the gDNA of P. aeruginosa strains was quantified using LC-MS/MS. TBCF, CF isolate; TBCFΔ*cp*, *M.PaeTBCFII* deletion mutant of TBCF; TBCFΔ*cp*::*cp*, *paeTBCFIIM* complementation of TBCFΔ*cp*. (b) *In vitro* enzyme activity analysis. DNA substrates containing recognition motifs were incubated with purified M.PaeTBCFII (CP) protein, and the 6mdA/dA ratio was estimated by the relative abundance of 6mdA normalized to that of dA. The abundance of each type of base was calculated from the peak area. Seq1~3-CP, DNA substrates containing the motif TRGANNNNNNTGC recognized by M.PaeTBCFII (CP); Seq1~3-CP were incubated with M.PaeTBCFII (CP) protein for reactions. −, DNA substrate was not incubated with MTase; +, DNA substrate was incubated with MTase. Data are means and standard deviations from three independent experiments. **, *P < *0.01; ***, *P < *0.001.

To investigate the function of putative DNA MTase M.PaeTBCFII, we generated a gene knockout mutant, TBCFΔ*paeTBCFIIM*, as well as its complemented strain TBCF*com*Δ*paeTBCFIIM*. Deletion or complementation of these DNA MTase genes did not lead to significant changes in growth under standard laboratory conditions (see Fig. S2 in the supplemental material). As M.PaeTBCFII was predicted to be a 6mA methylase, we then assessed the influence on 6mA modification in the genomic DNA using LC-MS/MS. We tested the ratio of 6mdA (*N*^6^-methyl-2′-deoxyadenosine) and dA (2′-deoxyadenosine) concentration in the genomic DNA of TBCF and *paeTBCFIIM* deletion and complemented strains. Standard 6mdA at 10 nM can be well separated and identified by the elution procedure in Table S2, and the peak time is 3.23 min, as shown in Fig. S3. Quantification results of methylated bases by LC-MS/MS showed that 6mdA content was approximately 0.7556% of the total adenines throughout the genome of wild-type TBCF. Deletion of the gene *paeTBCFIIM* drastically decreased the 6mdA/dA ratio in TBCFΔ*paeTBCFIIM* mutant compared to wild-type TBCF. The complemented strain TBCF*com*Δ*paeTBCFIIM* has an even higher 6mdA/dA ratio than the wild type (Fig. 1a; Fig. S3; also, see Table S3-1 at https://doi.org/10.5061/dryad.gmsbcc2rb). These results suggested that M.PaeTBCFII is the dominant *N*^6^-adenine DNA MTase in TBCF, which is consistent with the SMRT-seq analysis result.

10.1128/msystems.00434-22.2 TABLE S2 LC-MS/MS elution procedure. Download Table S2, DOCX file, 0.01 MB. Copyright © 2022 Han et al. 2022 Han et al. https://creativecommons.org/licenses/by/4.0/ This content is distributed under the terms of the Creative Commons Attribution 4.0 International license.

10.1128/msystems.00434-22.4 FIG S2 Growth curve of P. aeruginosa TBCF, mutants, and complemented strains at 37°C with aeration in LB broth. Download FIG S2, PDF file, 0.1 MB. Copyright © 2022 Han et al. 2022 Han et al. https://creativecommons.org/licenses/by/4.0/ This content is distributed under the terms of the Creative Commons Attribution 4.0 International license.

10.1128/msystems.00434-22.5 FIG S3 Mass spectra of *in vivo* MTase enzyme activity assays. Related to Fig. 1a and Table S3-1. Quantification of the 6mdA/dA ratio using LC-MS/MS analysis. Genomic DNA of TBCF, MTase mutants, and complemented strains was purified and enzymatically digested into single nucleosides, followed by LC-MS/MS detection. RT, retention time; AA, peak area. Download FIG S3, PDF file, 0.1 MB. Copyright © 2022 Han et al. 2022 Han et al. https://creativecommons.org/licenses/by/4.0/ This content is distributed under the terms of the Creative Commons Attribution 4.0 International license.

We also performed SMRT-seq on the Δ*paeTBCFIIM* deletion mutant and found a loss of adenine methylation within the motif TRGANNNNNNTGC throughout the genome (see Table S4 at https://doi.org/10.5061/dryad.gmsbcc2rb). The results further confirmed that the DNA adenine MTase M.PaeTBCFII specifically methylates the TRGANNNNNNTGC sequence motif.

### *In vitro* enzyme activity analysis of MTase using LC-MS/MS.

To assess the *in vitro* 6mA modification activity and specificity of the predicted MTase, M.PaeTBCFII protein was expressed and purified (Fig. S4). Three DNA fragments containing predicted methylation target motifs were amplified and allowed to react with the purified M.PaeTBCFII protein. The changes in 6mdA/dA were detected using LC-MS/MS. In the control experiment without the MTases, almost no methylation occurred. DNA modification capacity tests showed that the 6mdA/dA ratios of nucleic acid fragments listed in Appendix A (https://doi.org/10.5061/dryad.gmsbcc2rb) increased to various degrees. The results indicated that the levels of 6mA increased in the presence of the two MTases, with more obvious changes observed after the addition of M.PaeTBCFII (Fig. 1b; Fig. S5; also, see Table S3-2 at https://doi.org/10.5061/dryad.gmsbcc2rb). Taken together, the results showed that M.PaeTBCFII has a high *in vitro* 6mA modification activity.

10.1128/msystems.00434-22.6 FIG S4 MTases M.PaeTBCFII (CP) protein purification results. (a) SDS-PAGE analysis after washing on Ni-NTA column; (b) ÄKTA system results after washing on Superdex 200 molecular sieve columns; (c) SDS-PAGE analysis after washing on Superdex 200 molecular sieve columns. The red box indicates the sample with the correct molecular weight. Download FIG S4, PDF file, 0.3 MB. Copyright © 2022 Han et al. 2022 Han et al. https://creativecommons.org/licenses/by/4.0/ This content is distributed under the terms of the Creative Commons Attribution 4.0 International license.

10.1128/msystems.00434-22.7 FIG S5 Mass spectra of *in vitro* MTase enzyme activity assays. Example of Seq1-CP. DNA substrate containing methylation recognition motif was incubated with M.PaeTBCFII (CP) protein for reaction, and the degree of modifying activity was quantified using LC-MS/MS. RT, retention time; AA, peak area; ND, nondetectable; Seq1-CP, DNA substrate containing the recognition motif TRGANNNNNNTGC of CP; CP+ Seq1-CP, Seq1-CP incubated with M.PaeTBCFII protein for reaction. Download FIG S5, PDF file, 0.1 MB. Copyright © 2022 Han et al. 2022 Han et al. https://creativecommons.org/licenses/by/4.0/ This content is distributed under the terms of the Creative Commons Attribution 4.0 International license.

### RNA sequencing analysis revealed changes in transcriptional profiles of the MTase mutants.

**FIG 2:**
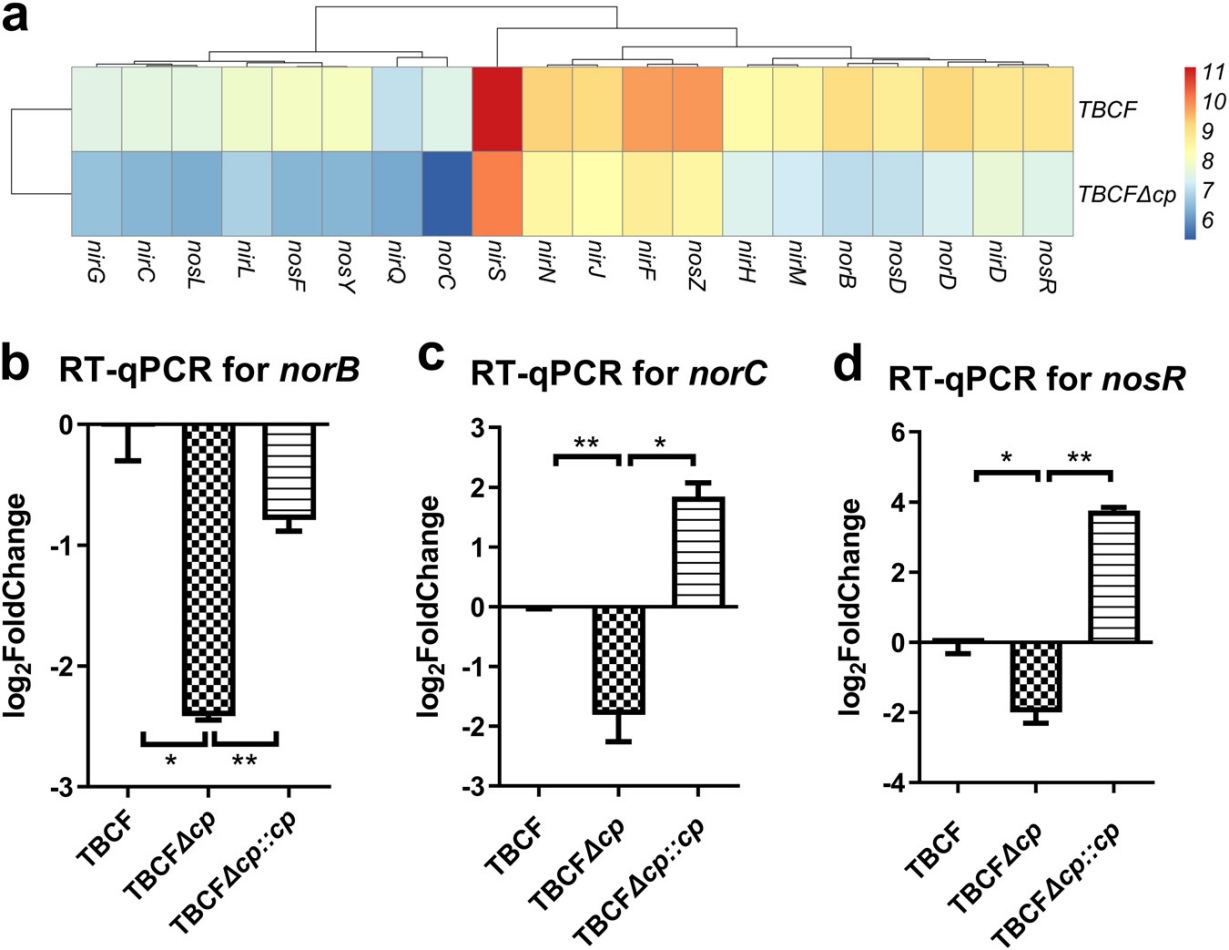
Transcription of the denitrification gene clusters of TBCF and the *paeTBCFIIM* deletion mutant. (a) Heat maps representing the expression profiles of indicated denitrification genes. The colored bars represent the expression levels on a log_2_ scale. (b to d) RT-qPCR results for TBCF, TBCFΔ*cp*, and TBCFΔ*cp*::*cp* for the denitrification-related genes *norB* (b), *norC* (c), and *nosR* (d). Each column presents the mean and SD for three biological replicates per group. *, *P < *0.05; **, *P < *0.01.

To study the regulatory effect of the MTases on the gene expression of TBCF, we performed transcriptome sequencing (RNA-seq) analysis and obtained differentially expressed genes (DEGs) between the wild type (TBCF) and the knockout mutant TBCFΔ*paeTBCFIIM* based on the selection criteria of a |log_2_(fold change [FC])| of >1.5 and an adjusted *P* value of <0.05. In contrast, 23 genes were downregulated and 19 genes were upregulated in TBCFΔ*paeTBCFIIM* compared to TBCF (Fig. S6a; also, see Table S5 at https://doi.org/10.5061/dryad.gmsbcc2rb) under the selected experimental conditions. Function enrichment analysis of the DEGs indicated that there were 4 pathways being enriched according to both GO and KEGG enrichment analysis (Fig. S6b; also, see Table S6 at https://doi.org/10.5061/dryad.gmsbcc2rb). The most notable observation was the downregulation of the operons for nitrogen metabolism, including *nirSMCFDL*, *norCBD*, and *nosRZDFYL*. These gene clusters encode nitrite reductase (NIR), nitric oxide reductase (NOR) and nitrous oxide reductase (NOS), respectively. The drop in the expression of *norC* (log_2_ FC = −3.0247) and *norB* (log_2_ FC = −3.0188), which encode NO reductase subunits C and B, were the most significant. In addition, the expression of *nosR*, encoding a key regulatory protein, was also significantly reduced (log_2_ FC= −2.0826) (Fig. 2b to d; also, see Table S5 at https://doi.org/10.5061/dryad.gmsbcc2rb).

10.1128/msystems.00434-22.8 FIG S6 RNA-seq data analysis. (a) Volcanic plot showing DEGs between TBCF (*n* = 3) and TBCFΔ*cp* (*n* = 3). The DEGs (|log_2_ FC| > 1.5 and adjusted *P* value < 0.05) are in red (upregulated) and blue (downregulated). Each dot represents one gene. (b) Bar plot showing pathways in both GO and KEGG enriched by the DEGs between TBCF and TBCFΔ*cp* (*P*  < 0.05). The *x* axis shows counts of genes, and the *y* axis shows the pathways. Darker bar color indicates a lower *P* value. Download FIG S6, PDF file, 0.1 MB. Copyright © 2022 Han et al. 2022 Han et al. https://creativecommons.org/licenses/by/4.0/ This content is distributed under the terms of the Creative Commons Attribution 4.0 International license.

The reduction in the expression of *norB*, *norC*, and *nosR* was verified by reverse transcription-quantitative PCR (RT-qPCR). We used the complemented strains as controls and *rpsL* as a housekeeping gene. Expression of these three genes was confirmed to be significantly downregulated in TBCFΔ*paeTBCFIIM*, and the complemented strain could reverse these gene expression levels. Interestingly, the expression of *norC* and *nosR* was upregulated in complemented strain TBCF*com*Δ*paeTBCFIIM* compared to the TBCF wild type (Fig. 2b to d), which suggests tight control of gene expression by M.PaeTBCFII.

### Target motifs of M.PaeTBCFII exist in differentially expressed genes.

**FIG 3:**
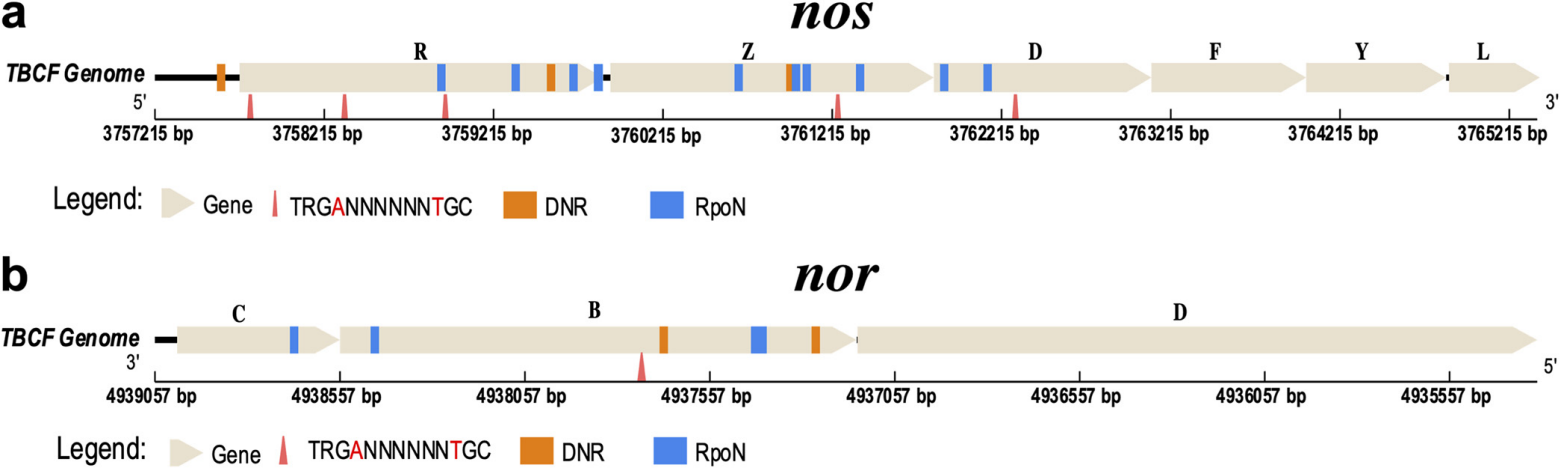
*In silico* analysis of the putative regulatory region of *nos* and *nor* operons. (a) Location diagram of M.PaeTBCFII methylation motifs (triangles), RpoN binding sites (blue rectangles), and DNR binding sites (orange rectangles) in *nos* genes (yellow arrow). (b) Location diagram of M.PaeTBCFII methylation motifs and RpoN and DNR binding sites in *norCB* genes. BEDTools, the FIMO tool, and in-house shell scripts were used to annotate methylated genes.

To investigate how DNA MTase influence the expression of DEGs, we searched for M.PaeTBCFII target motifs in promoters or coding regions of all the 42 DEGs identified. Twelve motifs were found, with 11 being located in the gene body and 1 in the promoter region. Of the 42 DEGs, 8 harbored one or more M.PaeTBCFII recognition motifs, including *nosR*, *nosZ*, *nosD*, *norB*, and *nirF* (see Table S5 at https://doi.org/10.5061/dryad.gmsbcc2rb). Three M.PaeTBCFII recognition motifs are located in *nosR*. Among them, one is close to the ATG translation start codon (distance of 58 nucleotides [nt]). There is one M.PaeTBCFII recognition motif in the *norB* coding region. Previous studies showed that transcription factor DNR and sigma factor RpoN affect the expression of *nos*, *nir*, and *nor* (19–21). Therefore, we searched for recognition motifs of DNR and RpoN in the gene regulatory and coding sequences. We found many RpoN and DNR binding sites near the methylation motifs (see Tables S5, S8, and S9 at https://doi.org/10.5061/dryad.gmsbcc2rb; Fig. 3).

### MTase M.PaeTBCFII regulates intercellular survival of TBCF in NO-producing macrophages.

**FIG 4:**
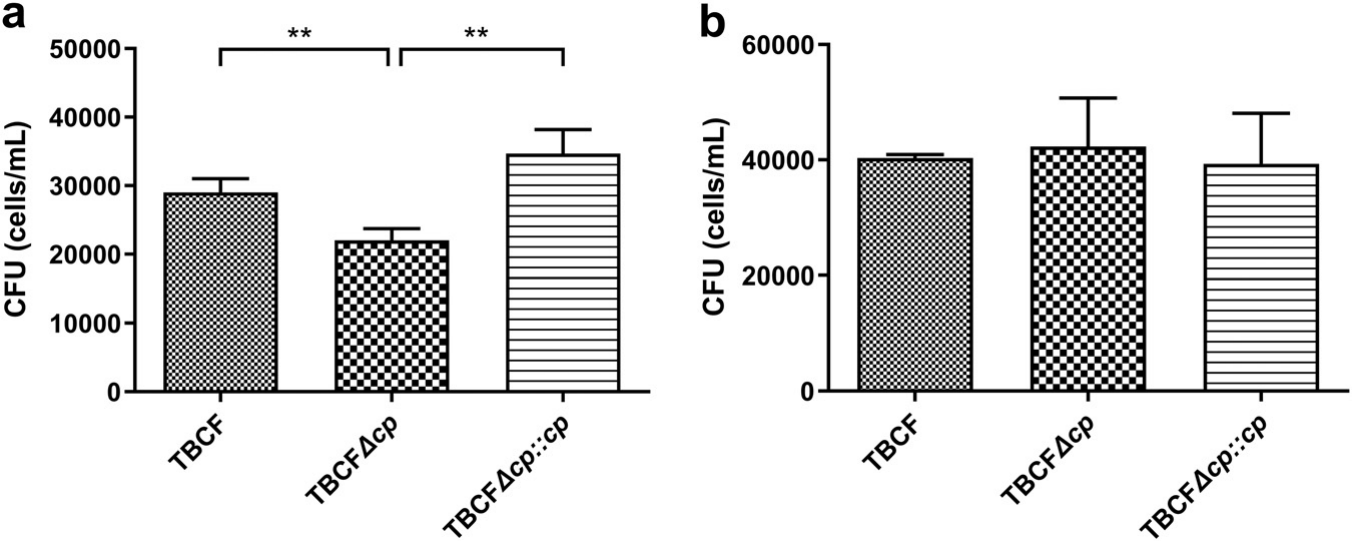
Intracellular survival of internalized P. aeruginosa. Numbers of CFU per well at 2 h are shown. (a) Bacteria were internalized by RAW264.7 macrophages treated with LPS 24 h prior to bacterial infection. (b) The iNOS inhibitor l-NMMA (1 mM) was added after washing to remove extracellular bacteria. Data are means and SD from at least three independent experiments performed in triplicate. **, *P* < 0.01, determined by independent two-sample *t* test.

To investigate the impacts of DNA methylation on the intracellular survival of TBCF, we performed a macrophage infection assay. To induce the NO production via inducible NO synthase (iNOS), RAW264.7 macrophages were pretreated with lipopolysaccharide (LPS) 24 h before the experiment (22). After uptake by macrophages, the number of viable TBCFΔ*paeTBCFIIM* cells was significantly lower than that of TBCF cells after 2 h of killing. Furthermore, no significant differences were observed between the number of viable TBCF*com*Δ*paeTBCFIIM* and TBCF cells (Fig. 4a). However, upon incubation with a 1 mM concentration of the NOS inhibitor N(G)-monomethyl-l-arginine (l-NMMA) during continuous infection, no significant difference was observed among the strains (Fig. 4b). This result indicated that TBCFΔ*paeTBCFIIM* is more vulnerable than TBCF to the intracellular environment of LPS-activated NO-producing macrophages.

### M.PaeTBCFII regulates virulence of TBCF in a G. mellonella infection model.

**FIG 5:**
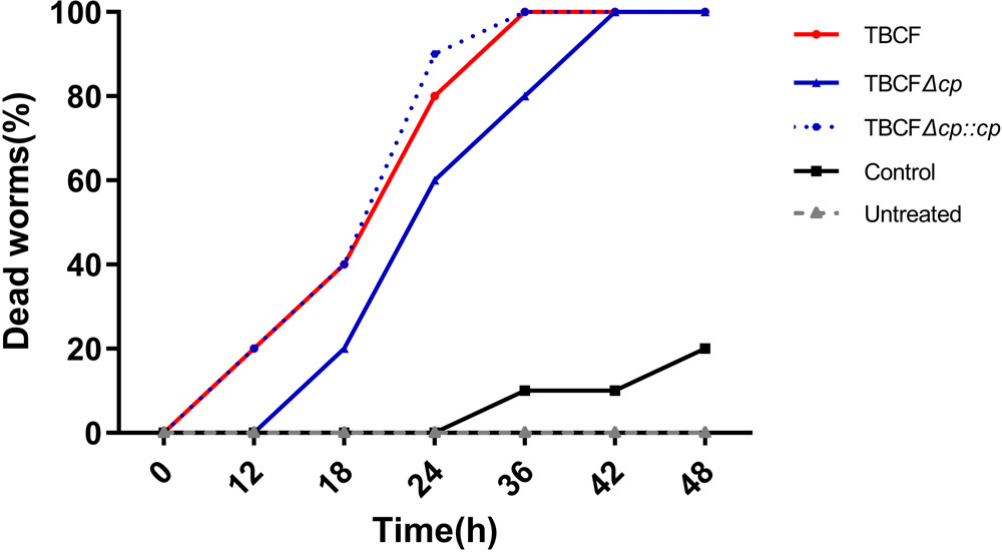
Relative death rates of P. aeruginosa-infected G. mellonella larvae. Larvae were treated with TBCF (red), TBCFΔ*cp* (blue), TBCFΔ*cp*::*cp* (blue with dashed line), or 5 μL sterile PBS control (black) or left untreated (gray dashed line). The experiment was done in replicates on different days, and the percentage of larvae that were dead at each time was plotted against the time of incubation.

To test the regulatory effect of the MTase M.PaeTBCFII on the virulence of TBCF, we analyzed and compared the relative survival rates of G. mellonella larvae after infection with TBCF, the M.PaeTBCFII mutant, and its complemented strain. At 12 h after infection, no dead larvae were observed in the TBCFΔ*paeTBCFIIM* group, while the mortality rates of G. mellonella larvae in the TBCF group and complemented-strain group were both 20%. At 36 h after infection, 20% of the larvae in TBCFΔ*paeTBCFIIM* group survived, while all larvae in the wild-type group and complemented-strain group had died. We also analyzed relative survival rates in a G. mellonella infection model, and the M.PaeTBCFII-deficient TBCF mutant exhibited a marginally but significantly decreased virulence phenotype (50% lethal dose [LD_50_], 22.5 h) compared with the TBCF wild type (LD_50_, 19.5 h), and the result for the complemented strain (LD_50_, 19.2 h) returned to the wild-type level. Our results showed that the deletion of *paeTBCFIIM* in TBCF attenuates the virulence of the strain, which is consistent with the results of the intracellular-survival experiment (Fig. 5).

## DISCUSSION

P. aeruginosa is one of the most common nosocomial pathogens and is a classic model organism for studying bacterial gene expression regulation. P. aeruginosa TBCF10839 is a chronically host-adapted strain isolated from a cystic fibrosis (CF) patient (18, 23), and its special antiphagocytosis characteristic has aroused research interest. In this study, we systematically characterized the methylome of TBCF and discovered the role of its DNA MTase in transcriptional regulation and virulence. We found that 6mA modification is the main type of DNA methylation and that M.PaeTBCFII is the dominating enzyme responsible for 6mA modification in TBCF.

The M.PaeTBCFII-deficient TBCF mutant has a much lower expression level of certain genes involved in nitrogen metabolism, such as *nir*, *nos*, and *nor* operons. NIR, NOR, and NOS are components of the denitrification process, catalyzing the reaction cascade NO_2_^−^ → NO → N_2_O → N_2_. Denitrification is a critical process regulating the removal of bioavailable nitrogen from natural and human-altered systems. Denitrification performance is critical for microbial versatility in response to different selection pressures, including bacterial infection, where large amounts of NO can be generated by iNOS from macrophages (24–26). In addition to being observed in bacteria, denitrification has also been found in archaea and in the mitochondria of fungi. Pseudomonads represent one of the largest groups of the denitrifying bacteria within a single genus, favoring their use as model organisms (27–29).

Of note, we showed here that NOR genes, *norCBD*, are the most significantly downregulated genes in a M.PaeTBCFII-deficient mutant. A survival test in macrophages and a virulence test using the G. mellonella infection model revealed that the M.PaeTBCFII-deficient mutant has lower viability in NO-producing macrophages and decreased virulence to G. mellonella larvae. NO and its homologues play essential roles in the host defense against pathogen invasion, while NO reductase (NOR) is a key factor for pathogens to withstand NO pressure and host immunity (24–26). NOR widely exists in denitrifying bacteria like Pseudomonas and other microorganisms (30). The NOR enzyme family allows bacteria to bypass the NO-related immune defense in the host and convert NO into nitrous oxide (N_2_O), which has lower cytotoxicity, and this is conducive to bacterial proliferation in the host (26, 30). For example, pyrite nitric oxide reductase (flavodiiron NOR [FNOR]) is an enzyme that catalyzes the reduction of NO to N_2_O. It widely exists in desulfurized *Vibrio*, intestinal Salmonella, and some Escherichia coli strains and helps the pathogen to survive under nitrous stress in anaerobic environments (31). Kakishima et al. found that wild-type P. aeruginosa PAO1 survived longer than *norCBD* mutants in NO-producing macrophages, and they therefore proposed that the NOR in P. aeruginosa functions as a detoxifying enzyme contributing to its intracellular survival and ability to resist host defense mechanisms (32).

M.PaeTBCFII has the recognition motif TRG**A**NNNNNN**T**GC (modification sites are in bold). The results of RNA-seq analysis and motif identification showed that not all of the DEGs harbored a methylation motif in their promoter or coding region. This indicates that there are impacts from secondary effects on gene transcription. One interesting candidate for the mediation of these indirect effects is *nosR*, which is a known denitrification regulator (33, 34). We found three methylation motifs in the coding region of *nosR*; one of them is close to the ATG translation start codon (distance < 100 nt), transcription of which was reduced in the M.PaeTBCFII deletion strain. Of note, one M.PaeTBCFII target motif was also found in the coding region of *norB*, which encodes nitric oxide reductase subunit B*. In silico* analysis found sigma factor RpoN and transcription factor DNR binding sites located near the M.PaeTBCFII target motifs in *nosR* and *norB*. RpoN (σ^54^) was reported to have a repressive effect on the transcription of nitrogen-regulated genes, such as *nirS*, *norC*, and *nosR* (20, 35, 36). Inversely, DNR positively regulates *nosR* transcription. DNR belongs to the cyclic AMP receptor protein (CRP)-FNR transcription factor superfamily. When the concentration of intracellular NO increases, NO binds to DNR heme complex and regulates the expression of *nirS*, *norCB*, and *nos* (19). Many previous studies focused on the discovery of methylation sites in upstream promoter regions of genes (5, 15, 37). In our study, we frequently found methylation target motifs in the coding regions of several differently expressed genes, and the RpoN binding sites were found close to these methylation target motifs. A combined chromatin immunoprecipitation sequencing (ChIP-seq)–RNA-seq assay revealed *in vivo* binding sites of RpoN in the P. aeruginosa PAO1 genome. Notably, over 80% of the ChIP peaks were inside the coding region (21). The results of our study suggested that modification sites located in the gene body may also play important roles in epigenetic regulation by impeding the binding of important transacting factors. Based on this, we speculated that methylated motifs in promoter or coding regions of these genes affect binding of transacting factors, leading to shifts in gene expression. However, this hypothesis should be validated experimentally.

This study explored the regulatory effect of the orphan DNA MTase M.PaeTBCFII in P. aeruginosa TBCF10839. To the best of our knowledge, this is the first report that a DNA MTase regulates the transcription of denitrification genes represented by NOR and affects antiphagocytic ability in bacteria. This research not only deepens the understanding of the role of DNA MTase in transcriptional regulation in P. aeruginosa but also provides a theoretical foundation for the in-depth study of the molecular mechanism of the epigenetic regulation of the denitrification process, virulence, and host-pathogen interaction. To find homologous proteins in Pseudomonas and other bacteria, an NCBI BLASTX search was performed using the M.PaeTBCFII sequence. The results showed that homologs of M.PaeTBCFII are frequently found in P. aeruginosa isolates (among the top 30 hits, 27 isolates have a homolog with 100% coverage and >97% identity) (see Table S7 at https://doi.org/10.5061/dryad.gmsbcc2rb). In addition, many other pathogens, including species of Pseudomonas, *Vibrio*, Klebsiella, and Escherichia, encode a protein that is >80% identical (Fig. S7). Interestingly, the M.PaeTBCFII recognition motifs are also found in nitrogen metabolism genes (for example, *nosR* and *norB*) in Pseudomonas strains with M.PaeTBCFII homologs (Fig. S8). Thus, our findings that DNA methylation regulates denitrification gene expression in P. aeruginosa provides a new insight into the epigenetic regulation of the denitrification process in P. aeruginosa and other organisms under different conditions.

10.1128/msystems.00434-22.9 FIG S7 Phylogenetic tree of strains containing the top 100 M.PaeTBCFII (CP) homologs. The phylogenetic tree was constructed using the neighbor-joining method with bootstrap values of 1,000 replications. Visualization was implemented in iTOL (https://itol.embl.de/). Blue, Pseudomonas; red, *Vibrio*; green, Klebsiella; yellow: Escherichia. Download FIG S7, PDF file, 0.7 MB. Copyright © 2022 Han et al. 2022 Han et al. https://creativecommons.org/licenses/by/4.0/ This content is distributed under the terms of the Creative Commons Attribution 4.0 International license.

10.1128/msystems.00434-22.10 FIG S8 Multiple sequence alignment shows methylation recognition motifs of M.PaeTBCFII (CP) in *nosR* (a) or *norB* (b) from 10 different Pseudomonas strains. Gene nucleotide sequences were aligned with Muscle v 3.8 (48). M.PaeTBCFII recognition motifs are indicated in yellow, as well as 6mA modification sites (red underlines) and the ATG translation start codon (green). The number behind each line is the distance from the last nucleotide in the line to the translation start codon. The number after each strain name shows the sequence length of the gene; for example, “P. aeruginosa_IMP-13/1-2148” indicates that the length of *nosR* in P. aeruginosa IMP-13 is 2,148 bp. Download FIG S8, PDF file, 0.9 MB. Copyright © 2022 Han et al. 2022 Han et al. https://creativecommons.org/licenses/by/4.0/ This content is distributed under the terms of the Creative Commons Attribution 4.0 International license.

## MATERIALS AND METHODS

### Strains and culture conditions.

All strains and plasmids used in this study are listed in Table 2. Bacteria were grown in Luria-Bertani (LB) liquid medium or on agar (1.5% Bacto agar) plates supplemented with appropriate antibiotics at 37°C with aeration unless stated otherwise. Carbenicillin (60 μg/mL), chloramphenicol (6 μg/mL), or kanamycin (50 μg/mL) was used for Escherichia coli strains. Tetracycline (60 μg/mL) was used for complemented TBCF strains.

**TABLE 2:** Bacterial strains and plasmids in this study

Plasmid or strain	Description^*a*^	Abbreviation	Reference or source
Plasmids			
pK18	Small mobilizable vector, Gm^r^, sucrose sensitive (*sacB*)		49
RK600	Cm^r^ ColE1 *oriV* RK2 *mob*^+^ *tra*^+^; helper plasmid in triparental matings		50
pET28a	Contains a fused N-terminal 6×His tag, a MBP tag, and a TEV protease recognition sequence		51
mini-CTX1	Tc^r^; self-proficient integration vector with *tet*, V-FRT-attPMCS, *ori*, *int*, and *oriT*		52
Strains			
E. coli			
TOP10	F^−^ *mcrA* Δ(*mrr*-*hsd* RMS-*mcrBC*) ϕ80*lacZ* Δ*M15* Δ*lacX74 recA1 ara*Δ*139* Δ(*ara*-*leu*)*7697 galU galK rpsL* (Str^r^) *endA1 nupG*		Tiangen
BL21(DE3)	F^−^ *ompT hsdSB*(r_B_^−^ m_B_^−^) *gal dcm* (DE3）		TransGen
P. aeruginosa			
TBCF10839	CF isolate	TBCF	23
TBCFΔ*paeTBCFIIM*	*paeTBCFIIM* deletion mutant of TBCF	TBCFΔ*cp*	This study
TBCF*com*Δ*paeTBCFIIM*	*paeTBCFIIM* complementation of TBCFΔ*paeTBCFIIM*	TBCFΔ*cp*::*cp*	This study

a Gm^r^, gentamicin resistance; Cm^r^, chloramphenicol resistance; Str^r^, streptomycin resistance; Tc^r^, tetracycline resistance; MBP, maltose-binding protein; TEV, tobacco etch virus.

### Genomic extraction and sequencing.

TBCF wild type and MTase knockout strains were cultured to mid-log phase. Genomic DNA of the strains was extracted using an AxyPrep bacterial genomic DNA miniprep kit (Corning, New York, NY, USA) and Mabio bacterial DNA extraction minikits (Mabio), respectively, using the manufacturers’ standard protocols. SMRT-seq was performed using the method described in reference (38). Basically, DNA was fragmented with g-Tubes (Covaris) and end repaired to prepare SMRTbell DNA template libraries. Genomic sequencing was performed on the Pacific Biosciences RSII sequencer (PacBio, Menlo Park, CA, USA) according to standard protocols.

### Methylome analysis.

The quality of SMRT-seq reads were assessed using SequelTools version 1.3.4 (-p a -n 40 -t Q -u) (39). SMRT-seq reads were assembled into a complete genome using the HGAP4 pipeline of SMRTLink software v9.0 with default settings. The TBCF genome was annotated by Prokka v1.14.6 (the “–proteins PAO1.faa” option was used to ensure that gene naming was consistent with the good-quality PAO1 reference genome) (40). DNA methylation analysis was performed using the base modification analysis and motif analysis applications of SMRTLink software v9.0. The complete genome sequence was uploaded into the SMRT portal as the reference sequence. A default modification quality value (QV) score of 30 (corresponding to a *P* value of 0.001) was used to call the modified bases. Restriction modification system genes were predicted and assigned to identified recognition motifs with REBASE (41).

### Construction of MTase deletion mutants.

The in-frame deletion mutagenesis of MTase was performed through two-step allelic exchange (42). For gene *paeTBCFIIM* (encoding the putative DNA MTase M.PaeTBCFII in TBCF) in-frame deletion, the upstream and downstream DNA fragments of *paeTBCFIIM* were amplified with two pairs of primers, *CPF1/CPR1* and *CPF2/CPR2*, respectively. PCR products were purified using a HiPure PCR Pure minikit (Magen) and were ligated to the HindIII- and EcoRI-digested suicide vector PK18 using Gibson Assembly master mix (New England Biolabs [NEB]). After verification of the sequence, the suicide plasmid was transferred from E. coli Top10 (donor strain) to P. aeruginosa TBCF (recipient strain) by conjugal mating with the help of the pRK600 vector. Gentamicin was used for selection of the first homologous recombinants. Selected colonies were then streaked onto LB agar with 20% sucrose to select for the second homologous recombinants. The *paeTBCFIIM* deletion mutant TBCFΔ*paeTBCFIIM* (also called TBCFΔ*cp*) was verified by PCR using primers *CPF3* and *CPR3*. All primers used are listed in Table S1.

10.1128/msystems.00434-22.1 TABLE S1 Primers used for protein purification, mutant strains construction, methylation motif-containing sequences amplification, and RT-qPCR. Download Table S1, DOCX file, 0.02 MB. Copyright © 2022 Han et al. 2022 Han et al. https://creativecommons.org/licenses/by/4.0/ This content is distributed under the terms of the Creative Commons Attribution 4.0 International license.

### Construction of MTase complemented strains.

To ensure that phenotypes were caused solely by the deletion of DNA MTase, the deletion mutant was complemented. For *paeTBCFIIM* complementation, the promoter region (525 bp before the translation initiation codon ATG) and the full-length gene fragment were PCR amplified using the primers *cCPF1* and *cCPR1*. The PCR products were purified using a HiPure PCR Pure minikit (Magen) and were ligated to the HindIII- and BamHI-digested vector mini-CTX1 using Gibson Assembly master mix (NEB). After verification of the sequence using the primers *cCPF2* and *cCPR2*, the plasmid was transferred from E. coli Top10 (donor strain) to TBCFΔ*paeTBCFIIM* (recipient strain) by conjugal mating with the help of the pRK600 vector and was selected on LB agar plates containing 100 μg/mL tetracycline. The *paeTBCFIIM*-complemented strain TBCF*com*Δ*paeTBCFIIM* (also called TBCFΔ*cp*::*cp*) was verified by PCR using the primers *cCPF2* and *cCPR2.* All primers are listed in Table S1.

### Quantification of modified bases in gDNA by LC-MS/MS.

The method for quantification of modified bases in genomic DNA (gDNA) was adapted from a previous study (5). Briefly, 50 μL gDNA each from P. aeruginosa TBCF, MTase deletion mutants, and complemented strains (about 1 μg) was denatured at 100°C for 5 min, followed by chilling on ice for 2 min and digestion using nuclease P1 (100,000 U/mL; NEB no. M0660) at a ratio of 1 μL in 5 μL 10× nuclease P1 reaction buffer (NEB no. M0660) at 37°C for 1 h. One microliter of antarctic phosphatase (5,000 U/mL; NEB no. M0289) and 6 μL antarctic phosphatase reaction buffer (NEB no. M0289) were then added, and incubation continued at 37°C overnight. The final solution was analyzed using LC-MS/MS. The LC-MS/MS elution process is shown in Table S2.

The LC-MS/MS measurements were performed on a Shimadzu Nexera high-performance liquid chromatography (HPLC) system (Shimadzu) coupled with a QTrap5500 triple-quadrupole mass spectrometer equipped with an electrospray ionization (ESI) source (Thermo Scientific Q Exactive). DNA-derived deoxynucleosides were separated using a 50- by 4.6-mm Zorbax Eclipse XDB C_18_ reverse-phase (RP) HPLC column (1.8-μm particle size) (Agilent, Santa Clara, CA). In addition, a 2-μm ColumnSaver filter (Supelco, Bellefonte, PA) and a C_18_ RP SecurityGuard (Phenomenex, Aschaffenburg, Germany) were connected in front of the separation column. HPLC-grade water (Acros, Belgium) with 0.1% formic acid (CNW, Germany) was used as solvent A, while HPLC-grade methanol (Merck, Germany) and 0.1% formic acid were used as solvent B. The gradient started at 95% solvent A for 0.3 min, followed by a linear increase of solvent B up to 50% until 7.1 min. Solvent B concentration was held for another 1 min at 50% and decreased to 5% until 8.2 min. Equilibration with solvent A at 95% was performed until 11.2 min. The flow rate was 0.4 mL/min. The injection volume was 10 μL.

### Purification of MTase.

The open reading frame (ORF) encoding MTase was amplified by PCR using TBCF genomic DNA as the template. The PCR product was purified using a HiPure PCR Pure minikit (Magen, Guangzhou, China) and was ligated to the BamHI- and XhoI-digested vector pET28a using Gibson Assembly master mix (NEB, USA) to yield pET28a-MTase. After verification of the sequence, the plasmid was extracted using a TIANprep mini-plasmid kit (Tiangen, Beijing, China) and was transformed into E. coli BL21(DE3). Briefly, after overnight culturing in 10 mL LB broth with 50 μg/mL kanamycin, the culture was transferred into 1 L of LB broth with 50 μg/mL kanamycin and was grown to an optical density at 600 nm (OD_600_) of 0.6 at 37°C with constant shaking at 220 rpm. Isopropyl-d-1-thiogalactopyranoside (IPTG) was added at a final concentration of 0.5 mM to induce protein expression at 18°C for 16 h. After centrifuging at 4°C and 5,000 rpm for 15 min, the pellet was resuspended in 100 mL of buffer A (150 mM NaCl, 20 mM Tris-HCl [pH 8.0], 10 mM imidazole and 1 mM phenylmethylsulfonyl fluoride [PMSF]). Then cells were lysed using sonication (Sonics, USA) with an interval of 6 s for 1.5 h on ice in the cold room. The sonicated suspension was centrifuged at 4°C and 12,000 rpm for 1 h and was then filtered using a 0.22-μm-pore filter. The filtrate was loaded into a nickel-nitrilotriacetic acid (NTA) column (GE, USA). The Ni-NTA column was washed five times with buffer B (150 mM NaCl, 20 mM Tris-HCl [pH 8.0], 1 mM imidazole); then, a 30 mL gradient of 10 to 300 mM imidazole was prepared in buffer B, and the washed fractions were collected. Sodium dodecyl sulfate-polyacrylamide gel electrophoresis (SDS-PAGE) was used to verify the molecular weight of the target protein using the collected fractions. Proteins were concentrated by ultracentrifugation (Eppendorf, Germany) at 4°C at 4,000 rpm until the volume was less than 500 μL. MTases were then purified using a Superdex 200 molecular sieve column (GE, USA) on an ÄKTA system following the manufacturer’s protocol and stored at −80°C until use.

### *In vitro* MTase activity assays.

One-microgram DNA duplexes containing the motif TRGANNNNNNTGC (PCR products of TBCF purified with a HiPure PCR pure minikit [Magen]; primers are listed in Table S1 and sequences are listed in Appendix A at https://doi.org/10.5061/dryad.gmsbcc2rb) were incubated with 1 μM purified M.PaeTBCFII (a putative DNA MTase in TBCF) protein at 30°C for 1 h in reaction buffer (20 mM Tris–HCl, pH 8.0; 100 mM KCl; 0.1 mM EDTA; 3 mM β-mercaptoethanol; 80 μM SAM). The quantified modified bases of methylated products were tested by LC-MS/MS, with the parameters described above.

### RNA sequencing and analysis.

The RNA preparation and comparative analysis of gene expression were carried out using previously described protocols (38). P. aeruginosa TBCF and mutants were cultured to mid-log phase. Total RNA extraction and purification was performed for 3 biological replicates of each strain. RNA sequencing was performed on the Illumina NovaSeq 6000 sequencing platform, generating 150-bp paired-end reads. Raw reads of the samples were preprocessed and analyzed using RSEM v1.3.1 with the unique mapping setting (–bowtie-m), using the P. aeruginosa TBCF genome as the reference. Differential gene expression was analyzed using DESeq2 with the selection criteria of an absolute log_2_ fold change of >1.5 and an adjusted *P* value of <0.05. GO and KEGG enrichment analysis of significantly regulated genes was performed on the DAVID bioinformatics database v2021 (43). Volcano plots and heat maps were drawn using ggplot2 and pheatmap packages in R 4.1.2 software.

### Real-time qPCR verification.

Total RNAs of TBCF, mutants, and complemented strains were extracted using an RNeasy minikit (Qiagen, Germany) and were DNA decontaminated using DNase (Qiagen, Germany). cDNA was amplified using Hifair III first-strand cDNA synthesis supermix (Yeasen). RT-qPCR assays were performed using the Hieff qPCR SYBR green master mix (Yeasen) and qPCR systems (Roche) according to the manufacturers’ instructions. RT-qPCR primers used are listed in Table S1. cDNA of each strain was diluted to 10 ng/μL. Ten microliters of the total PCR volume was used according to the manufacturer’s protocol. The following PCR protocol was used: one cycle at 50°C for 2 min and 95°C for 2 min, followed by 45 cycles at 95°C for 15 s, 57°C for 15 s, and 72°C for 60 s. LightCycler 96 software (Roche) was used for data analysis. The relative expression levels of target genes were calculated by normalizing against the expression of the housekeeping gene *rpsL.* The relative gene expression levels in TBCF and mutants were compared. The RT-qPCR experiment was performed in triplicate.

### Scanning for transcription factor- and sigma factor-binding motifs.

BEDTools and the FIMO tool were used to annotated methylated genes (44, 45). Transcription factor (TF)-binding and sigma factor-binding motifs near methylated bases in DEGs were searched using the command line motif scanner FIMO version 4.12.0. For each gene containing a methylated locus in an MTase target motif, the sequences of the whole gene and 500 bp upstream of the coding start site (putative promoter region) were extracted. The context sequences were combined into a multisequence FASTA file. FIMO was then run using each TF motif on the context FASTA file with a threshold *P* value of 0.01. The TF motifs were downloaded from collecTF (46).

### Growth assay.

Overnight cultures of P. aeruginosa TBCF10839 and mutants were diluted to an OD_600_ of 0.01 in fresh LB liquid medium for use as the inoculum. One hundred microliters of the inoculum was aliquoted into 96-well microtiter plate in triplicate and incubated statically for 24 h at 37°C for growth. OD_600_ values were recorded for 16 h in a Tecan Infinity Pro 200 microplate reader (Spark) for plotting of growth curves.

### Macrophage intracellular survival assay.

The method for macrophage intracellular survival assays was adapted from the protocol used by Kakishima et al. (32). RAW264.7 cells, a murine macrophage line, were maintained in Dulbecco’s modified Eagle’s medium (DMEM) supplemented with 10% (vol/vol) heat-inactivated fetal bovine serum (hiFBS), penicillin G (100 U/mL), and streptomycin (100 μg/mL), which constitutes complete medium, at 37°C in 95% (vol/vol) air–5% (vol/vol) CO_2._ RAW264.7 cells were inoculated into 24-well culture plates in complete medium with 10 μg/mL lipopolysaccharide (LPS) at a density of 4 × 10^8^/L for overnight incubation at 37°C. The medium was exchanged with 500 μL of fresh DMEM with 10% hiFBS, penicillin, and streptomycin prior to experiments. After that, TBCF, mutants, and complemented strains were seeded into macrophage cultures at a density of 4,000 CFU per well (multiplicity of infection [MOI] = 50). Cells were incubated at 37°C under 5% CO_2_ for 0.5 h to allow internalization. After internalization, cells were washed twice by pipetting using prewarmed phosphate-buffered saline (PBS) to remove extracellular bacteria. Then, 500 μL of fresh DMEM supplemented with 10% hiFBS was added to each well, followed by further incubation for 2 h at 37°C under 5% CO_2_ with or without 1 mM l-NMMA. Cells were then rinsed once with PBS and lysed with 200 μL of 0.1% Triton X-100. To enumerate the survival rate of intracellular bacteria, an appropriate dilution of the lysate was plated onto LB agar plates and incubated for CFU counting.

### G. mellonella larva infection model.

P. aeruginosa TBCF, mutants, and complemented strains were cultured overnight in LB liquid medium at 37°C and 220 rpm. The overnight cultures were diluted to 1:100 in the fresh LB broth and grown to an OD_600_ of ~0.8. Cultures were centrifuged, and pellets were resuspended in 10 mM MgSO_4_ to an OD_600_ of 0.1. Serial 10-fold dilutions were made in 10 mM MgSO_4_ supplemented with 0.5 mg of rifampin/mL to 5,000 CFU. Fifth-stage G. mellonella larvae that were bright and white without gray-black spots and with a limited mass from 50 mg to 350 mg were randomly placed in groups of 10 each. Five-microliter aliquots of the serial dilutions of bacterial cultures were injected into G. mellonella larvae with microliter syringes (Gaoge, China). A final concentration of approximately 10 μg of rifampin per gram of larva was added to prevent infection by natural bacterial flora on the surface of the larvae (47). Larvae were incubated in 10-cm plates at room temperature, and the number of dead larvae was recorded after infection. A larva was considered dead when it displayed no movement in response to touch.

### Phylogenetic analysis of DNA MTase.

The nucleotide sequence of DNA MTase was used to perform alignment in the protein database by NCBI BLASTX (https://blast.ncbi.nlm.nih.gov/Blast.cgi). A phylogenetic tree was constructed using the 100 most similar proteins, with the neighbor-joining method and bootstrap values of 1,000 replications. Then, visualization was implemented in the iTOL (https://itol.embl.de/). In addition, the closest neighbors of the DNA MTase were also searched using BLASTP analysis in REBASE.

### Conservation analysis of some denitrification genes in strains with M.PaeTBCFII homologs.

Nine Pseudomonas strains (including 7 Pseudomonas aeruginosa strains and 2 other Pseudomonas strains, which have MTase M.PaeTBCFII homologs with >90% identity) were selected to find potential methylated motifs in *nosR* and *norB*. Multiple sequence alignment was performed in MUSCLE v 3.8 (48) with default parameters.

### Data analysis and statistics.

Data are presented as means and standard deviations (SD). Unless otherwise specified, comparisons were made using an independent two-sample *t* test using SPSS 20. Statistical significance was determined using a *P* value of <0.05 or <0.01. Graphs were drawn using GraphPad prism 5.0.

### Data availability.

The data generated in this study are available upon request. The genome of P. aeruginosa strain TBCF is available at GenBank under accession number CP096207. SMRT-seq and RNA sequencing data for TBCF and TBCFΔ*paeTBCFIIM* (TBCFΔ*cp*) strains are available at NCBI’s Sequence Read Archive as part of BioProject PRJNA835892 and PRJNA830320. Some supplemental material items (Tables S3 to S9 and Appendix A) are provided in an online repository (https://doi.org/10.5061/dryad.gmsbcc2rb).
