# Supplementary figures and images for: DNA Methyltransferase Regulates Nitric Oxide Homeostasis and Virulence in a Chronically Adapted Pseudomonas aeruginosa Strain
Source: mSystems. 2022 Sep 15;7(5):e00434-22. doi: 10.1128/msystems.00434-22 (PMC9600465; doi:10.1128/msystems.00434-22)

a

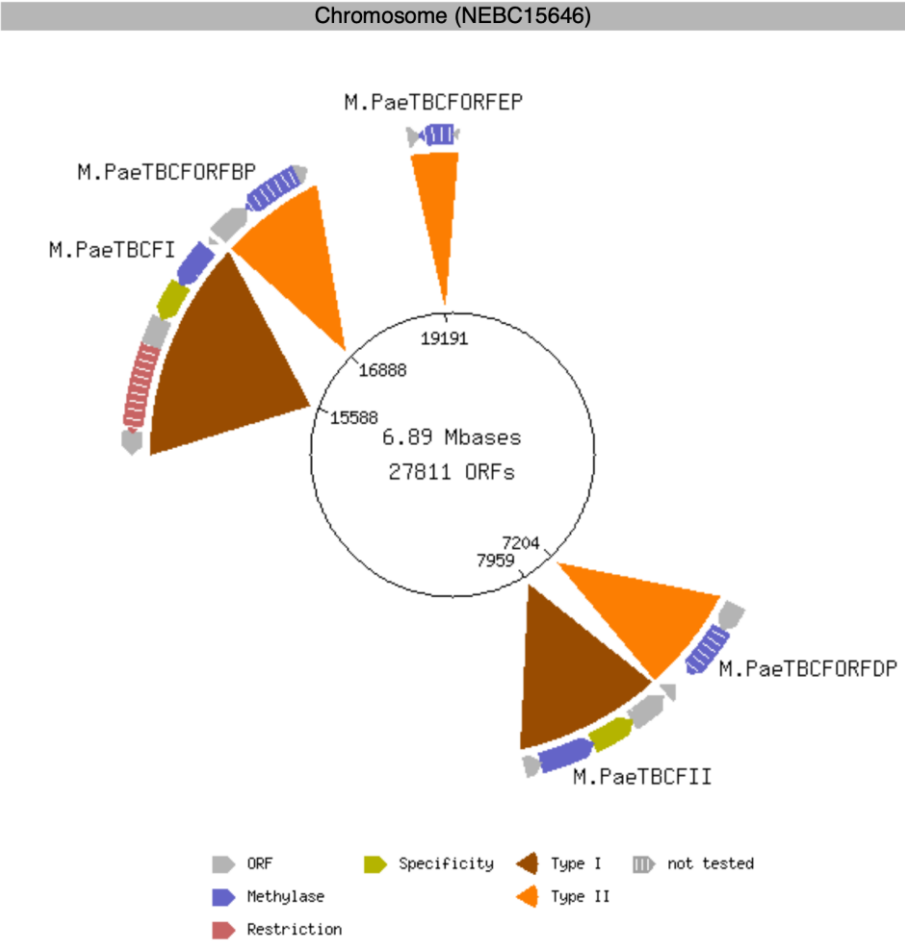

b

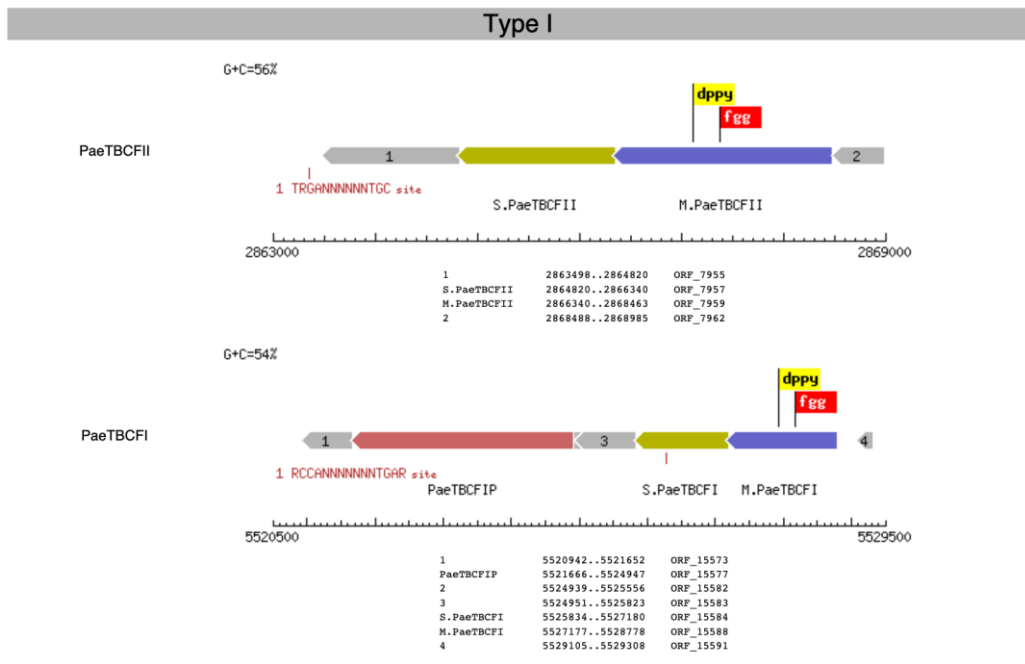

Supplement: FIG S1 [file msystems.00434-22-s0003.pdf]

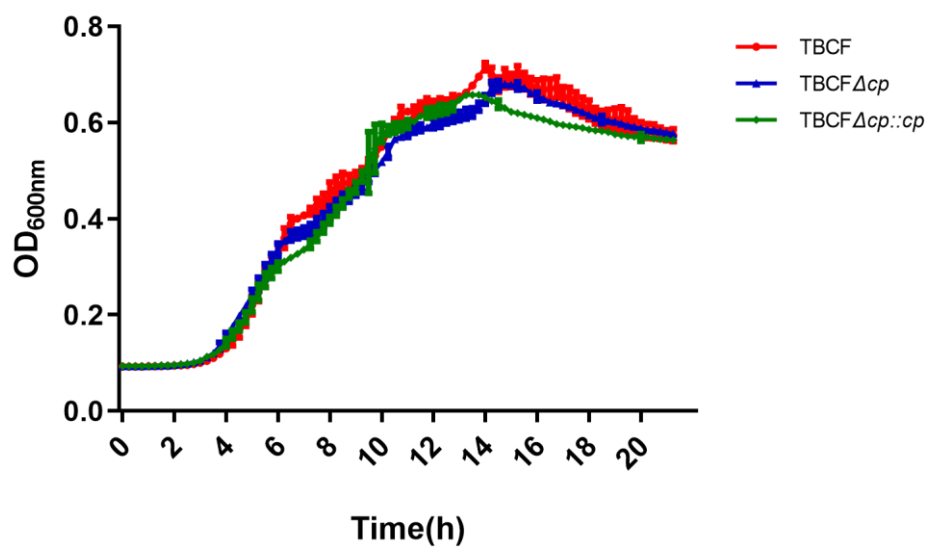

Supplement: FIG S2 [file msystems.00434-22-s0004.pdf]

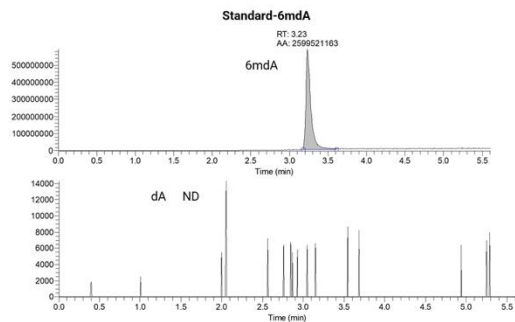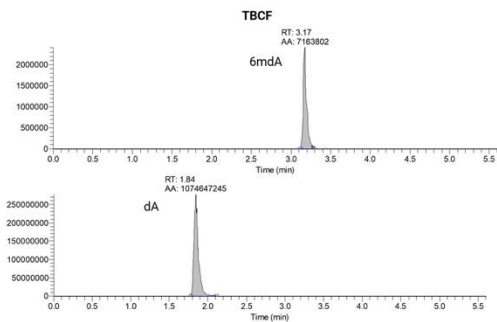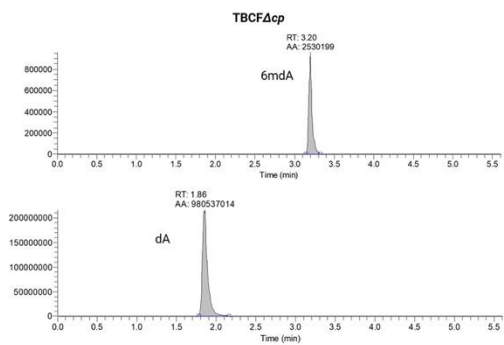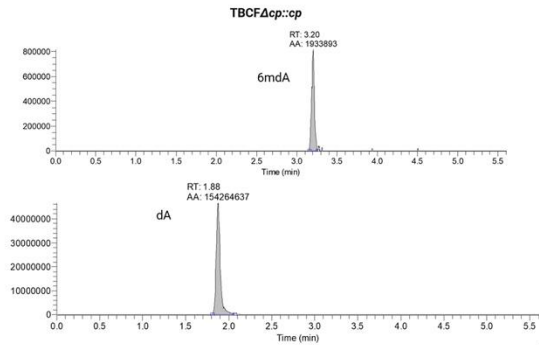

Supplement: FIG S3 [file msystems.00434-22-s0005.pdf]

### Seq1-CP

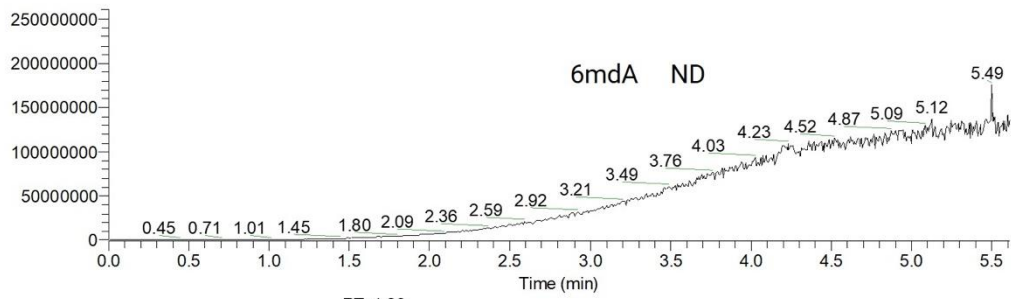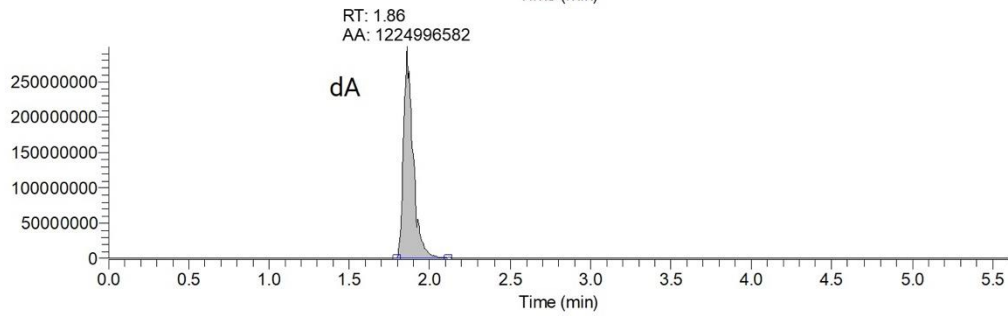

### CP+Seq1-CP

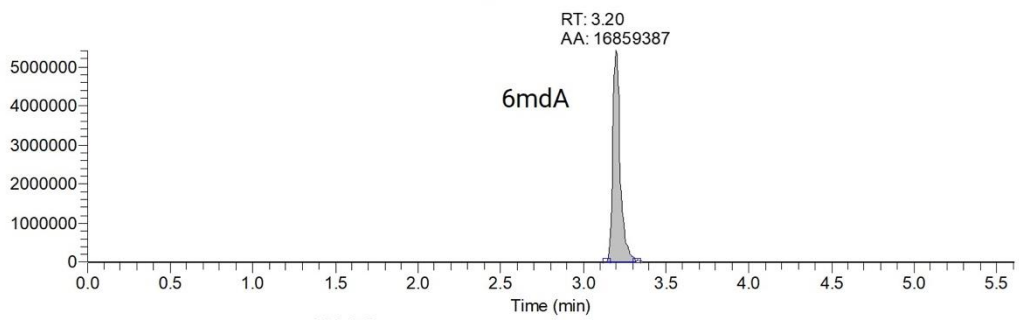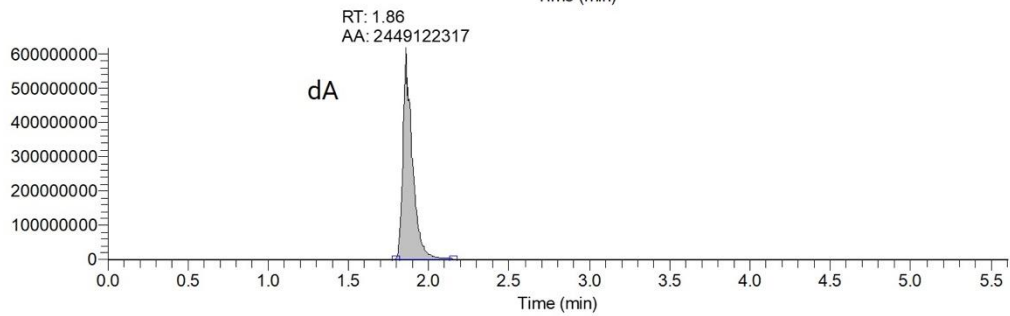

Supplement: FIG S5 [file msystems.00434-22-s0007.pdf]

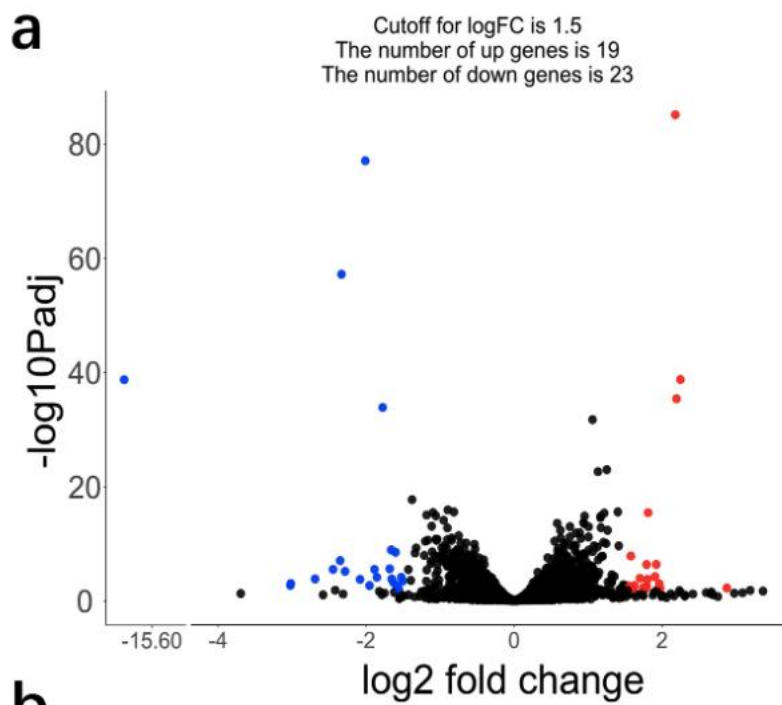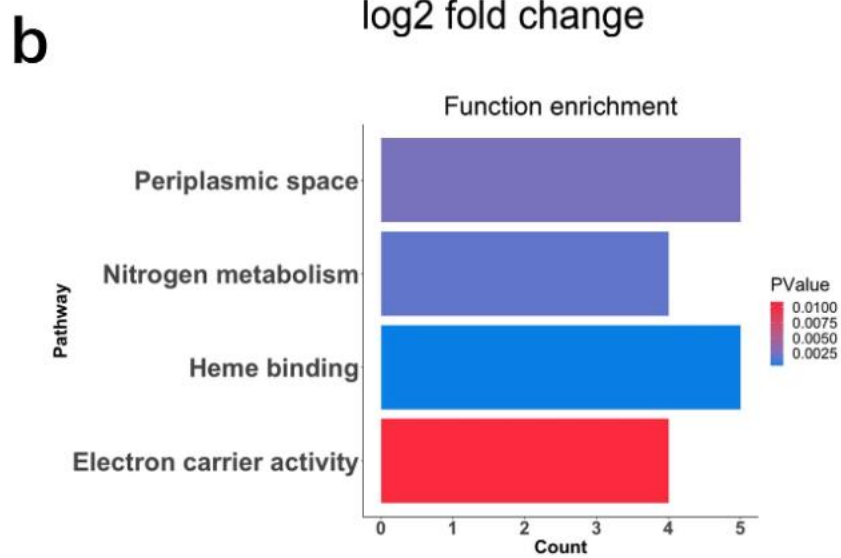

Supplement: FIG S6 [file msystems.00434-22-s0008.pdf]

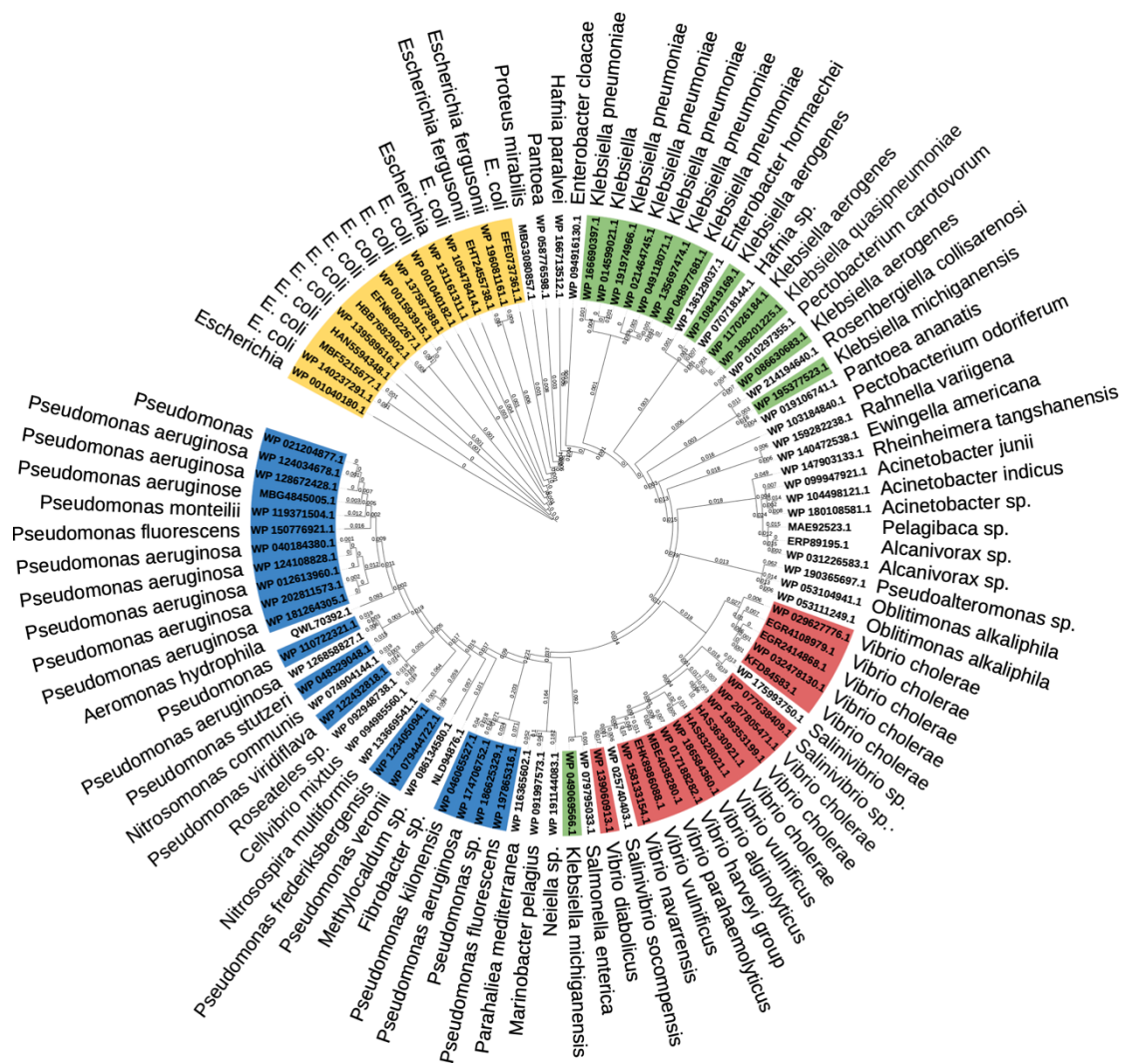

Supplement: FIG S7 [file msystems.00434-22-s0009.pdf]
