# Supplementary material for: DNA Methyltransferase Regulates Nitric Oxide Homeostasis and Virulence in a Chronically Adapted Pseudomonas aeruginosa Strain
Source: mSystems. 2022 Sep 15;7(5):e00434-22. doi: 10.1128/msystems.00434-22 (PMC9600465; doi:10.1128/msystems.00434-22)
Supplement: TABLE S2 [file msystems.00434-22-s0002.docx]

**Table S2** LC-MS/MS elution procedure

| Time point | Solvent A | Solvent B |
| --- | --- | --- |
| 0 | 95% | 5% |
| 0.3 | 95% | 5% |
| 7.1 | 50% | 50% |
| 8.1 | 50% | 50% |
| 8.2 | 95% | 5% |
| 11.2 | 95% | 5% |

Solvent A, HPLC-grade water with 0.1% formic acid; Solvent B, HPLC-grade methanol with 0.1% formic acid.
