# Supplementary material for: DNA Methyltransferase Regulates Nitric Oxide Homeostasis and Virulence in a Chronically Adapted Pseudomonas aeruginosa Strain
Source: mSystems. 2022 Sep 15;7(5):e00434-22. doi: 10.1128/msystems.00434-22 (PMC9600465; doi:10.1128/msystems.00434-22)
Supplement: FIG S4 [file msystems.00434-22-s0006.pdf]

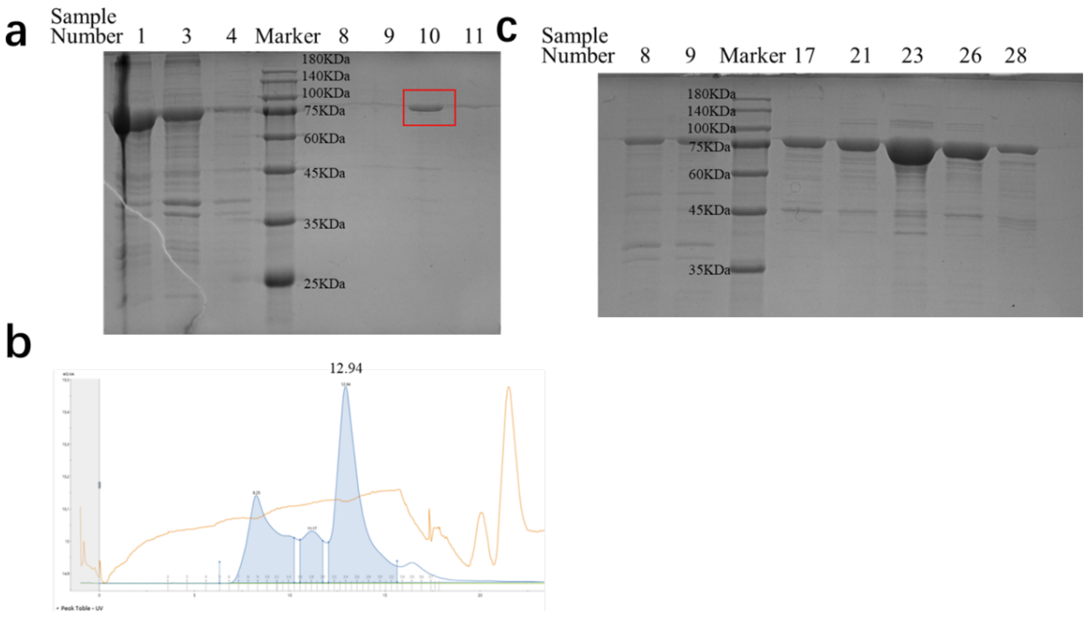

Figure S4. MTases CP protein purification results. a-c are the results of CP protein purification. (a) SDS-PAGE analysis of samples after washed by Ni-NTA column; (b) AKATA system results after washed by superdex200 molecular sieve column; (c) SDS-PAGE analysis of samples after washed by superdex200 molecular sieve column. The red box indicates the sample with the correct molecular weight.
