# Supplementary material for: DNA Methyltransferase Regulates Nitric Oxide Homeostasis and Virulence in a Chronically Adapted Pseudomonas aeruginosa Strain
Source: mSystems. 2022 Sep 15;7(5):e00434-22. doi: 10.1128/msystems.00434-22 (PMC9600465; doi:10.1128/msystems.00434-22)
Supplement: FIG S8 [file msystems.00434-22-s0010.pdf]

a

```
MTase_CP_Motif/1-39                ------------------------------------------------TRGANNNNNNTG   12
P.aeruginosa_TBCF-NosR/1-2148 ATGCGCGCCCTTCGTTTTTCCGCCGGATCGTGGCGGTGTGTCTTCGCCTGGATGCTCCTG   60
P.aeruginosa_IMP-13/1-2148    ATGCGCGCCCTTCGTTTTTCCGCCGGATCGTGGCGGTGTGTCTTCGCCTGGATGCTCCTG   60
P.aeruginosa_HS9/1-2148       ATGCGCGCCCTTCGTTTTTCCGCCGGATCGTGGCGGTGTGTCTTCGCCTGGATGCTCCTG   60
P.aeruginosa_FA-HZ1/1-2148    ATGCGCGCCCTTCGTTTTTCCGCCGGATCGTGGCGGTGTGTCTTCGCCTGGATGCTCCTG   60
P.aeruginosa_KRP1/1-2148      ATGCGCGCCCTTCGTTTTTCCGCCGGATCGTGGCGGTGTGTCTTCGCCTGGATGCTCCTG   60
P.aeruginosa_LES431/1-2148    ATGCGCGCCCTTCGTTTTTCCGCCGGATCGTGGCGGTGTGTCTTCGCCTGGATGCTCCTG   60
P.aeruginosa_LESB65/1-2148    ATGCGCGCCCTTCGTTTTTCCGCCGGATCGTGGCGGTGTGTCTTCGCCTGGATGCTCCTG   60
P.aeruginosa_6762/1-2148      ATGCGCGCCCTTCGTTTTTCCGCCGGATCGTGGCGGTGTGTCTTCGCCTGGATGCTCCTG   60
P.veronii_R02/1-1846          ------------------------------------------------------------   0
P.stutzeri_SLG510A3/1-1920    ------------------------------------------------------------   0

MTase_CP_Motif/1-39           C-----------------------------------------------------------   13
P.aeruginosa_TBCF-NosR/1-2148 CTGGTCGGCCTCGCCGCCCAGGGTGCCGAACTCAGCGATTTGGAGCGCCTGCGCATCGCC   120
P.aeruginosa_IMP-13/1-2148    CTGGTCGGCCTCGCCGCCCAGGGTGCCGAACTCAGCGAGTTGGAGCGTCTGCGCATCGCC   120
P.aeruginosa_HS9/1-2148       CTGGTCGGCCTCGCCGCCCAGGGTGCCGAACTCAGCGAGCTGGAGCGTCTGCGCATCGCC   120
P.aeruginosa_FA-HZ1/1-2148    CTGGTCGGCCTCGCCGCCCAGGGTGCCGAACTCAGCGAGCTGGAGCGTCTGCGCATCGCC   120
P.aeruginosa_KRP1/1-2148      CTGGTCGGCCTCGCCGCCCAGGGTGCCGAACTCAGCGAGCTGGAGCGTCTGCGCATCGCC   120
P.aeruginosa_LES431/1-2148    CTGGTCGGCCTCGCCGCCCAGGGTGCCGAACTCAGCGAGTTGGAGCGCCTGCGCATCGCC   120
P.aeruginosa_LESB65/1-2148    CTGGTCGGCCTCGCCGCCCAGGGTGCCGAACTCAGCGAGTTGGAGCGCCTGCGCATCGCC   120
P.aeruginosa_6762/1-2148      CTGGTCGGCCTCGCCGCCCAGGGTGCCGAACTCAGCAAGTTGGAGCGCCTGCGCATCGCC   120
P.veronii_R02/1-1846          ------------------------------------------------------------   0
P.stutzeri_SLG510A3/1-1920    ------------------------------------------------------------   0

...

MTase_CP_Motif/1-39           -------------------------TRGANNNNNNTGC----------------------   26
P.aeruginosa_TBCF-NosR/1-2148 ---CAACCGG-CAGCGACGGCGAGCTGGAGCGAATTGCTCGGCAACGGTGCGATCCGCCG   647
P.aeruginosa_IMP-13/1-2148    ---CAACCGG-CAGCGACCGCAAACTGGAGCGAATTGCTCGGCAACGGCGCGATCCGCCG   647
P.aeruginosa_HS9/1-2148       ---CAACCGG-CAGCGACGGCGAGCTGGAGCGAATTGCTCGGCAACGGCGCGATCCGCCG   647
P.aeruginosa_FA-HZ1/1-2148    ---CAACCGG-CAGCGACGGCGAGCTGGAGCGAATTGCTCGGCAACGGCGCGATCCGCCG   647
P.aeruginosa_KRP1/1-2148      ---CAACCGG-CAGCGACGGCGAGCTGGAGCGAATTGCTCGGCAACGGCGCGATCCGCCG   647
P.aeruginosa_LES431/1-2148    ---CAACCGG-CAGCGACGGCGAGCTGGAGCGAATTGCTCGGCAACGGCGCGATCCGCCG   647
P.aeruginosa_LESB65/1-2148    ---CAACCGG-CAGCGACGGCGAGCTGGAGCGAATTGCTCGGCAACGGCGCGATCCGCCG   647
P.aeruginosa_6762/1-2148      ---CAACCGG-CAGCGACGGCGAGCTGGAGCGAATTGCTCGGCAACGGCGCGATCCGCCG   647
P.veronii_R02/1-1846          -ACCGACCGGTTTGACCCCGCCACCTGGGCACAATTGACCGGCAATGGCGCGATCCGCCG   441
P.stutzeri_SLG510A3/1-1920    CGTCTACCAG-C-----CGGCCAACTGGACCGAGCTGACCGGCAGTGGCGCGATCCGGCG   447

...

MTase_CP_Motif/1-39           ----------------------TRGANNNNNNTGC-------------------------   39
P.aeruginosa_TBCF-NosR/1-2148 ACGGCCGAGGAACTGGCCGCCCTGGAAGAGGCTGCCCGGCCGCTGTGGCTGCGGGTCTGG   1245
P.aeruginosa_IMP-13/1-2148    ACCGCGGAGGAACTGGCCGCCCTGGAAGAGGCTGCCCGGCCGCTGTGGCTGCGGGTCTGG   1245
P.aeruginosa_HS9/1-2148       ACGGCCGAGGAACTGGCCGCCCTGGAAGAGGCCGCCCGGCCGCTGTGGCTGCGGGTCTGG   1245
P.aeruginosa_FA-HZ1/1-2148    ACGGCCGAGGAACTGGCCGCCCTGGAAGAGGCCGCCCGGCCGCTGTGGCTGCGGGTCTGG   1245
P.aeruginosa_KRP1/1-2148      ACGGCCGAGGAACTGGCCGCCCTGGAAGAGGCCGCCCGGCCGCTGTGGCTGCGGGTCTGG   1245
P.aeruginosa_LES431/1-2148    ACGGCCGAGGAACTGGCCGCCCTGGAAGAGGCTGCCCGGCCGCTGTGGCTGCGGGTCTGG   1245
P.aeruginosa_LESB65/1-2148    ACGGCCGAGGAACTGGCCGCCCTGGAAGAGGCTGCCCGGCCGCTGTGGCTGCGGGTCTGG   1245
P.aeruginosa_6762/1-2148      ACGGCCGAGGAACTGGCCGCCCTGGAAGAGGCTGCCCGGCCGCTGTGGCTGCGGGTCTGG   1245
P.veronii_R02/1-1846          ACCGCCGCCGAACAAGCCGCGCTGGATGAGGCCAGCCGCCCACTGTGGTTGAGCATCTGG   994
P.stutzeri_SLG510A3/1-1920    ACCGCCGAGGAACTGGCTGCCATTGAGGAAGCCAATCGGCCGATGTGGGTCAACATCTGG   1045

...
```

b

```
...

MTase_CP_Motif/1-13           ---TGGATCGGCGTGC--------------------------------------------   13
P.aeruginosa_TBCF-NorB/1-1398 TTCTGGATCGGCGTGCCCGGCTACTGGCTGTGGCTCGGCTCGGTATTCTCGGCGCTGGAG   840
P.aeruginosa_LESB65/1-1399    TTCTGGATCGGCGTGCCCGGCTACTGGCTGTGGCTCGGCTCGGTATTCTCGGCGCTGGAG   840
P.aeruginosa_LES431/1-1399    TTCTGGATCGGCGTGCCCGGCTACTGGCTGTGGCTCGGCTCGGTATTCTCGGCGCTGGAG   840
P.aeruginosa_6762/1-1399      TTCTGGATCGGCGTGCCCGGCTACTGGCTGTGGCTCGGCTCGGTATTCTCGGCGCTGGAG   840
P.aeruginosa_IMP-13/1-1399    TTCTGGATCGGCGTGCCCGGCTACTGGCTGTGGCTCGGCTCGGTATTCTCGGCGCTGGAG   840
P.aeruginosa_HS9/1-1398       TTCTGGATCGGCGTGCCCGGCTACTGGCTGTGGCTCGGCTCGGTATTCTCGGCGCTGGAG   840
P.aeruginosa_KRP1/1-1399      TTCTGGATCGGCGTGCCCGGCTACTGGCTGTGGCTCGGCTCGGTATTCTCGGCGCTGGAG   840
P.aeruginosa_FA-HZ1/1-1399    TTCTGGATCGGCGTGCCCGGCTACTGGCTGTGGCTCGGCTCGGTATTCTCGGCGCTGGAG   840
P.veronii_R02/1-1266          TTCTGGATCGGCGCACCCGAGGTGTGGCTGTGGGTCGGCTCGATCTTCTCGGCCCTGGAG   820
P.stutzeri_SLG510A3/1-1356    TTCTGGATCGGCGCACCGACCGTGTGGCTGTGGCTGGGCTCGATCTTCTCCGCCCTTGAG   820

...
```
