# Supplementary material for: DNA Methyltransferase Regulates Nitric Oxide Homeostasis and Virulence in a Chronically Adapted Pseudomonas aeruginosa Strain
Source: mSystems. 2022 Sep 15;7(5):e00434-22. doi: 10.1128/msystems.00434-22 (PMC9600465; doi:10.1128/msystems.00434-22)
Supplement: TABLE S1 [file msystems.00434-22-s0001.docx]

**Table S1. Primes used in this study.** Primers used for protein purification, mutant strains construction, methylation motif-containing sequences amplification, as well as RT-qPCR.

| *Name* | Sequence (5’-3’) | Application |
| --- | --- | --- |
| **Protein purification** | | |
| *pET28a-CPF* | CGCGGATCCATGGCAACCGAACAAACACT | For MTase CP purification |
| *pET28a-CPR* | CCGCTCGAGTCACTCACCCCGCTTGTACG | For MTase CP purification |
| **Construction of MTase deletion mutants** | | |
| *CPF1* | AgctcggtacccgggGGATCCCTTTGTCGCCGATGTATTTT | For MTase CP deletion |
| *CPR1* | GGAAAGAAGAACCCATGGCAGAGTGATGTTTGCAAAACGA | For MTase CP deletion |
| *CPF2* | TCGTTTTGCAAACATCACTCTGCCATGGGTTCTTCTTTCC | For MTase CP deletion |
| *CPR2* | CgacggccagtgccaaagcttGATCAACCTCGACCAGAGAT | For MTase CP deletion |
| *CPF3* | CCAAGACATCCGCTGCATAA | For the confirmation of MTase CP deletion |
| *CPR3* | ACCGAGATGGACTACAGCAT | For the confirmation of MTase CP deletion |
| **Construction of MTase complementary strains** | | |
| *cCPF1* | GTCGACGGTATCGATAAGCTTGCGATATCGTTTTGCAAACA | For MTase CP complementation |
| *cCPR1* | CGCTCTAGAACTAGTGGATCCTACACAGGAAAGAAGAACCC | For MTase CP complementation |
| *cCPF2* | TCTTGTCGAGATCATTCGGC | For the confirmation of MTase CP complementation |
| *cCPR2* | CAAGCCATCCGCGCCAATTA | For the confirmation of MTase CP complementation |
| **Amplification of sequences that contain methylation motif** | | |
| *1F* | GTTCGATACAGGCGTAGACC | For the purification of motif contains “TRGANNNNNNTGC” site |
| *1R* | ATCGCGATCAAGCTCTCCCC | For the purification of motif contains “TRGANNNNNNTGC” site |
| *2F* | TGCAGACGGTAGCCGTGATC | For the purification of motif contains “TRGANNNNNNTGC” site |
| *2R* | TCGCTGCTAACCAGCACCCG | For the purification of motif contains “TRGANNNNNNTGC” site |
| *3F* | TGAACCGACTGATCCGCTCA | For the purification of motif contains “TRGANNNNNNTGC” site |
| *3R* | GTGAAGGTCTTGCCGCCGAT | For the purification of motif contains “TRGANNNNNNTGC” site |
| **Primers for RT-qPCR** | | |
| *norBF* | CAGCGGCTATATCACCCAGG | For the confirmation of gene *norB* variation |
| *norBR* | TGGATGGATTGCAGGACACC | For the confirmation of gene *norB* variation |
| *norCF* | CGCGTTCCTCATCTCGATCA | For the confirmation of gene *norC* variation |
| *norCR* | TTGTTCTGTGCCACCAGGTT | For the confirmation of gene *norC* variation |
| *nosRF* | AGTACACCTGCAACGTCTGG | For the confirmation of gene *nosR* variation |
| *nosRR* | TTCACCAGGTCGACGAACAG | For the confirmation of gene *nosR* variation |
| *rpsLF* | AAGCGCATGGTCGACAAGA | For the amplification of gene *rpsL* |
| *rpsLR* | CGACCCTGCTTACGGTCTTT | For the amplification of gene *rpsL* |
